# A Review of Non-Invasive Techniques to Detect and Predict Localised Muscle Fatigue

**DOI:** 10.3390/s110403545

**Published:** 2011-03-24

**Authors:** Mohamed R. Al-Mulla, Francisco Sepulveda, Martin Colley

**Affiliations:** School of Computer Science and Electronic Engineering, University of Essex, Colchester, UK; E-Mails: fsepulv@essex.ac.uk (F.S.); martin@essex.ac.uk (M.C.)

**Keywords:** muscle fatigue, sEMG, feature extraction, classification

## Abstract

Muscle fatigue is an established area of research and various types of muscle fatigue have been investigated in order to fully understand the condition. This paper gives an overview of the various non-invasive techniques available for use in automated fatigue detection, such as mechanomyography, electromyography, near-infrared spectroscopy and ultrasound for both isometric and non-isometric contractions. Various signal analysis methods are compared by illustrating their applicability in real-time settings. This paper will be of interest to researchers who wish to select the most appropriate methodology for research on muscle fatigue detection or prediction, or for the development of devices that can be used in, e.g., sports scenarios to improve performance or prevent injury. To date, research on localised muscle fatigue focuses mainly on the clinical side. There is very little research carried out on the implementation of detecting/predicting fatigue using an autonomous system, although recent research on automating the process of localised muscle fatigue detection/prediction shows promising results.

## 1. Introduction

Muscle fatigue is a long lasting (several hours) reduction of the ability to contract and exert force. Generally, localised muscle fatigue occurs after a prolonged, relatively strong muscle activity. Due to the variability of the muscle characteristics from person to person there is no simple function of muscle load and timing which defines a precise muscle fatigue threshold [1]. Localised muscle fatigue can sometimes be beneficial in promoting muscle growth (as seen in bodybuilders) but it is usually harmful, causing serious injury when the level of fatigue is high. Muscles that are fatigued absorb less energy before they are stretched to such a degree it causes injuries [2]. In muscle fatigue research, fatigue is often related to the inability to reach a set level of maximum voluntary contraction (MVC) force, meaning that fatigue is related to an event or a time instant [3]. Myoelectric manifestations of fatigue can be detected by measuring electric current in the muscle and refer to changes in signal frequency and amplitude and in the muscle conduction velocity (CV), while the mechanical factors relate to a loss in the exerted force [4].

The detection and classification of muscle fatigue adds important information to the fields of human-computer interactions, sport injuries and performance, ergonomics and prosthetics. An automated system that will predict and detect when fatigue occurs is especially useful in sports related scenarios, where fatigue can lead to injury. An automated system will guide the user in his training by acting as a warning device before fatigue sets in while maintaining an optimal fatigue state, thus promoting improvements and avoiding unnecessary strain on the muscle so as to prevent injury. This system can also be applied in occupational health and ergonomics, in particular where there is a risk of work-related musculoskeletal disorders. Localised muscle fatigue in the work place may cause injury, for example, if a task causes elevated static muscle activity [5]. An automated system can predict when the muscle is fatiguing and hence avoid injury. Similarly, in ergonomics, a system like this can aid in the correction of posture problems before the occurrence of muscle strain or injuries.

When muscle activation occurs to lift a load, the muscle shortens and contracts [6]. Concentric contraction (*i.e.*, shortening of the muscle) will only occur if the load on the muscle is less than the tetanic tension that the muscle produces. Isometric contraction involves activation of the muscles but the muscle length does not change. When muscles are exercised they are sometimes active while lengthening; this is known as an eccentric contraction and it occurs when the subject walks or places an object on the floor. In the latter, the fall of the object is controlled by the active arm flexors. When non-isometric or dynamic contractions are said to be performed, dynamic exercise is being performed, where all of the three above mentioned contractions are present. This means that the muscles are constantly shortening and lengthening as the exercise is carried out.

Current research tends to focus on two classes of localised muscle fatigue: Non-Fatigue and Fatigue. Fatigue relates to the onset of fatigue during a muscle contraction, while Non-Fatigue is the state of the muscle during the contraction that occurs before the onset of fatigue. However, Al-Mulla *et al.* [7] suggested an additional, third class of fatigue: Transition-to-Fatigue. Their research highlights that attempting to predict the onset of the Fatigue class at the time at which it occurs is insufficient since then the predictive nature of the measurement is inherently lost. Rather, once the onset of Transition-to-Fatigue is detected, what follows is a progressive process until fatigue onset. In the first stage of fatigue, Non-Fatigue, the fresh muscle is able to exert its maximum force. Once the fresh muscle starts to fatigue, new recruitment of muscle fibres occurs. This is usually manifested as the onset of Transition-to Fatigue, where there is a sudden increase in motor unit action potential (MAUP) firing rate. After this increase, a progression of this state (Transition-to-Fatigue) is observed until the onset of Fatigue. This can be detected as there is a drop in myoelectric power emanating from the muscle due to loss in conduction velocity (CV) within the localised muscle.

In order to obtain comparable results in muscle fatigue research, certain criteria may be set for the selection of volunteer subjects. A number of health factors greatly influence the outcome and it is therefore important that the participating subjects are healthy. Any diseases or pre-existing conditions may influence the performance of the muscle groups, which will result in inconsistent findings for the same subject. Smoking and alcohol consumption are other influential factors. Wurst *et al.* [8] found that smokers experience greater peripheral muscle fatigue than non-smokers as smoke hampers oxygen delivery and, thus, mitochondrial function. Several studies have suggested that subjects should be advised to refrain from alcohol and caffeine on or before the test day [9]. Demura *et al.* [10] suggested that males may experience greater muscle fatigue than females for contractions at 40%–60% maximum voluntary contractions (MVC). In fatiguing contractions in young and old adults at different MVC, older subjects experience increased electromyographic activity levels [11]. This suggests that findings are influenced significantly by age and gender differences, such that the results from a twenty year old man and a sixty year old woman may not be comparable. Resting periods are often required for the subjects to ensure that the muscle group has not been recently exercised heavily, which can also influence the results.

**Figure 1.**
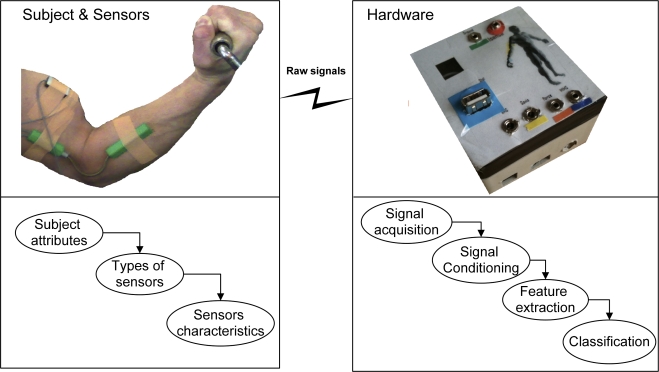
Experimental setup for an autonomous system to detect or predict fatigue.

A typical experiment in muscle fatigue research involves a subject performing a set task such as moving a given limb in a specified manner while sensors attached to the skin detect changes in signals arising from the movement. The acquired signal is then recorded and post-processed to reveal the characteristics of the muscle during that particular exercise. Subjects are normally healthy volunteers who have had the experiment explained to them so that they are able to give informed consent, which is approved by an ethics committee to their participation in the research study. A schematic of a typical experimental set-up is given in Figure 1.

Although much research on non-invasive localised muscle fatigue is based on surface electromyography (sEMG), this paper will firstly give a brief review of localised muscle fatigue acquisition techniques along with the various methodologies that are used to analyse the non-invasively acquired signals. This paper then goes on to discuss techniques for sEMG signal detection, processing, decomposition and classification along with their respective advantages and disadvantages. This paper will review the current state of the art in sEMG signal analysis techniques, such as the different methods of signal acquisition, whose features may be used for signal analysis, and finally, signal classification, so that the reader may identify the most appropriate methods that may be applied during clinical diagnosis, sports injury prevention, biomedical research, hardware implementation and end user applications.

## 2. Muscle Fatigue

There are no clearcut definitions of localised muscle fatigue and in the literature the different mechanisms of fatigue often overlap in their definitions because of the difficulty in isolating the different mechanisms of fatigue [12]; however, research has mainly focused on localised muscle fatigue as the process of a decline in the force during a sustained activity, which gives a definition of physiological fatigue as the inability to exert any more force or power [13]. Barry [13] argues that this definition indicates that fatigue occurs rapidly after the onset of a sustained exercise although the subject may be able to sustain the activity. Merletti and Parker [3] argue that muscle fatigue can be defined as an engineering approach to fatigue, where fatigue develops over time and is progressive, which defines muscle fatigue as all the physiological changes that occur in the muscle before reaching the inability to exert force. Physiological manifestations of fatigue relate to the exhaustion of the metabolic reserves in the contracting muscle. The amount of waste is increased, and the muscle has difficulty in continuing its task. As a result, the muscle fatigues due to the accumulation of lactic acid in the muscle tissue and the depletion of glycogen (stored glucose), which reduces the contractile properties of the muscle.

The neuromuscular mechanism of fatigue is related to changes in both the nerve system and the muscle causing it to fatigue, involving central fatigue (brain fatigue), fatigue in the neuromuscular junction and fatigue occurring in the muscle (peripheral fatigue) [3]. There are different kinds of muscle tissue in our body, however it is the skeletal muscle tissue, which is subject to voluntarily control, that is studied in investigations of localised muscle fatigue. Skeletal muscle tissue is composed of relatively large cells that are known as the muscle fibre, and which can be categorised depending on the speed of contractile twitch. All muscle tissues contain a mixture of both slow and fast twitching muscle fibres, in differing amounts. When muscle tissue contracts the muscle cells depolarise. When a motor neuron depolarises, an electrical current (the action potential, AP) is passed down the nerve fibre [14]. A motor unit (MU) consists of a single motor neuron located in the spinal cord and all of the muscle fibres that it innervates. When an MU fires, the electrical impulse (the AP) is carried down the motor neuron to the muscle. The area where the nerve and the muscle communicate is known as the neuromuscular junction or the motor endplate. After the AP is transmitted across the neuromuscular junction, it is elicited in all of the innervated muscle fibres of that particular MU. The result of all this electrical activity is known as a motor unit action potential (MUAP), which can be measured by EMG [14]. When a muscle fatigues, there is an increase in the recruitment of MUAPs.

Biomechanics is a study of internal and external forces acting on biological systems, such as the human skeletal muscles, and the effects produced by these forces. Biomechanical fatigue is often measured by speed, velocity and acceleration and is closely linked to kinematics and kinetics. In studies on muscle fatigue the joint angle and oscillations are recorded as indicators of fatigue [15–22].

In the literature on localised muscle fatigue researchers have identified that the mechanism responsible for fatigue is located in the exercising muscle (muscle mechanisms) or in the nervous system (brain mechanisms), where the former is termed peripheral fatigue and the latter central fatigue [23]. When muscles fatigue they contain both central and peripheral components, although their contribution to fatigue seems to be task dependent [24].

Peripheral fatigue relates to the ability of the muscle to perform physical work. When fatigue occurs, the normal functionality of the nerves and the muscles that are contracting are impaired, meaning that the muscle’s ability to exert force is declining due to the inability of the body to meet the increased energy demand in the contracting muscles. Peripheral fatigue is the most common case of physical fatigue. Central fatigue, on the other hand, relates to the central nervous system, involving connections from the brain to the nerves that are involved in muscle contraction. Central fatigue can be the result of changes in various neurotransmitters in the brain that are secondary to changes that occur in the body and brain. Generally, central fatigue can be described as the reduction in the nerve-based motor command that activates the muscles, which results in a decrease in the force output, in other words, total fatigue [25,26]. It has been argued that this reduction is a way to protect the muscles from injury if exercise is continued [27].

Both central and peripheral fatigue occur during sustained muscle activity where the effort is maximal [28], however, during sub-maximal effort more peripheral fatigue is present than central fatigue [29]. Nordlund *et al.* [30] suggested that intermittent maximal voluntary contractions are a useful model in the investigation of central and peripheral fatigue. In their study, which investigated peripheral and central contributions to fatigue during isometric intermittent maximal voluntary plantar flexions, they found that both central and peripheral mechanisms contributed to fatigue during repeated MVCs and that the level of voluntary activation and the initial plantar flexor strength influenced the development of peripheral, but not central, fatigue. Dimitrova *et al.* [31] developed new spectral indices of muscle fatigue and tested the interference of these indices on electromyography(EMG) signals during isometric contractions in the biceps brachii at various force levels. The indices proved to have higher sensitivity than traditional parameters (mean or median frequencies) but their sensitivity was dependent on the electrode placement. The authors argued that peripheral muscle fatigue is present when an increase in the values of the indices is observed, while a decrease in their values can be considered to be a sign of de-recruitment of fatigue, representing central fatigue.

### Muscle Fatigue Stages

In recent studies Almulla *et al.* [7,32,33] have identified new ways of classifying localised muscle fatigue, and in particular, they have identified an additional class of fatigue, termed Transition-to-Fatigue. This class exists after the Non-Fatigue class and before the onset of the Fatigue class. The third class of fatigue is a new concept in localised muscle fatigue research, which generally differentiate between only two classes of fatigue: Non-Fatigue and Fatigue. The identification of this third class will aid in the autonomous detection and prediction of muscle fatigue.

**Figure 2.**
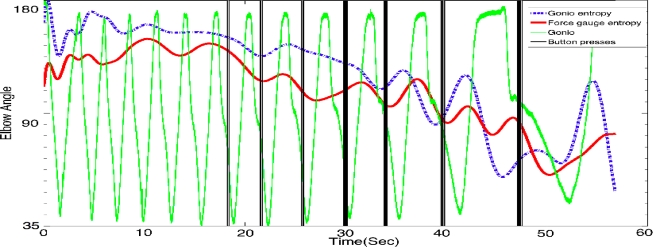
Movement aspects from one of the trials which aided in labelling the sEMG signal.

In previous research, Almulla *et al.* [7,33,34] labelled the sEMG signals using the recordings of the goniometer signal. This ensures that the sEMG classification in an autonomous system is correct, as the acquired sEMG signals is compared with the goniometer findings. To label the signals acquired from the subject into the three classes of fatigue (Non-Fatigue, Transition-to-Fatigue and Fatigue), Al-Mulla *et al.* used a fuzzy classifier (described in the classification section below) and the movement related aspects in a fatiguing contraction. For non-isometric contractions, the movement related aspects, such as speed of contractions, Moore-Garg Strain Index (button presses), elbow angle, arm vibration, force gauge, the entropy (function of probabilities in the signal) of elbow angle, and the entropy of force gauge, were considered as they are reliable indicators of fatigue in healthy subjects and in turn can correctly classify the sEMG signals [16–22]. The labelling was performed by human experts (a group composed of three experts concurring on the boundaries of the classes of fatigue) that used all the movement aspects of fatigue by referring to Table 1 as guide for labelling all the sEMG signals. Figure 2 shows, for illustration purposes, superimposed movement related signals for a single trial.

The identification of the Transition-to-Fatigue stage is important for the development of an automated system to predict localised muscle fatigue.

#### Discussion

Although most research on localised muscle fatigue divides the fatigue stages into two classes, Non-Fatigue and Fatigue, the Transition-to-Fatigue stage identified by Al-Mulla *et al.* is an important addition to the field, in particular for the development of real-time systems that automate the process of detecting and predicting fatigue. As researchers have identified two different mechanisms of fatigue, central and peripheral fatigue, this means that the fatigue stages have different mechanisms of fatigue that occur at various points during the fatigue stages, as Dimitrova *et al.* [31] have pointed out. Researchers have questioned how the increase in afferent feedback in response to changes in the metabolic and mechanical state of the muscle during a fatiguing contraction should be classified? Is this a central or a peripheral mechanism? [13]. It is clear that more research is needed on both central and peripheral fatigue to fully understand these mechanisms.

## 3. EMG and sEMG

EMG was first documented by Francesco Redi in 1666 when he found that a specialised muscle in the electric ray fish generates electricity. The term EMG appeared after the first recording of electrical muscle activity during voluntary contractions was made by Marey in 1890, who based this work on the discovery by Dubois-Raymonds in 1849 that such a recording would be possible. Since 1960, sEMG has been commonly applied in clinical research and Jasper, building an electromyograph, used it to demonstrate groundbreaking work on epilepsy and neurology. The field of EMG has been greatly influenced by Basmajian and De Luca [35]. Basmajian compiled all previous work on EMG in a book in 1965, which was later edited by De Luca, and this work has become a reference guide in the field of EMG. Although De Luca is probably the most influential worker on EMG, he warned that it is important to understand the short-comings of EMG, and in his paper on “The Use of sEMG in Biomechanics”, he argued that sEMG is such an easy tool to use in muscle physiology that it can easily be abused.

### 3.1. EMG Electrodes

EMG uses electrodes to detect electrical currents created in contracting muscles [36]. Although the idea that muscles generate electricity can be traced back to Francesco Redi’s work with electric eels circa 1666 [36], measuring this in a precise fashion is still considered to be a relatively new technology, and indeed one which is still under development.

The EMG signal is a complex and noisy signal, which generally should be filtered. The signal is described by its amplitude, frequency and phase as a function of time, amongst other parameters. EMG signals can be acquired both invasively, using needle electrodes, and non-invasively, by placing electrodes on the surface of the skin. The latter non-invasive case is termed surface EMG (sEMG) and is a common method used for acquiring the signals from muscle fatigue in both static and dynamic contractions. sEMG can be recorded from various parts of the body [37], but electrode placement is critical in ensuring reliable and repeatable results.

Other signal detection methods are discussed in the section below.

### 3.2. Electrode Types

Dimitrov *et al.* [38] studied various one- and two-dimensional multi-electrodes and their ability to provide the best selectivity and reduce crosstalk. They found that the new bitransversal double differentiating electrodes gave the best selectivity and the least crosstalk, in particular if placed above the end-plate region. They found that other electrodes (longitudinal double differential, transversal double differential and normal double differential) should be placed above the ends of the muscle generating the crosstalk. Linear arrays of electrodes have also been shown to give valuable information on neuromuscular systems, and are a useful tool in clinical research on muscle activities [39]. Alternatively, an array of electrodes can be placed longitudinally so that the electrodes cover the entire semi-length of the analysed fibres [31]. Such placement avoids the sensitivity of the electrodes when placed close to the end-plate region or the ends of the muscles. It is generally believed that future research will incorporate linear or two-dimensional arrays that will automatically detect the optimal channels and most reliable electrode placement location [40,41].

### 3.3. Electrode Placement and the Innervation Zone

The placement of the electrode influences the acquired signal since the measured electrical activity of the contracting muscle depends on the electrode location relative to the muscle. It is also important to place the electrodes in such a way to minimise crosstalk with signal from other nearby muscles, thus achieving reliable and stable surface electrode signals [42]. To minimise crosstalk, a threshold can be set for the background noise, and the position and the distance between the electrodes can be optimised. Gerdle *et al.* [43] argue that the optimal distance between electrodes is when the radius about the electrode where the signal amplitude is greatest, is larger than the standard deviation of the signal noise.

The area where nerve terminations and muscles connect is known as the innervation zone (IZ) [44]. There has been much research on the placement of electrodes for the avoidance of the IZ, in particular for studies on dynamic contractions, since it has been found that the EMG signal is affected if the electrodes are placed over the IZ [45]. The European SENIAM project attempted to reach consensus on electrode placement for 27 different muscles in the body to avoid the IZ, and resulted in a recommendation for electrode design and placement, recording and processing sEMG signals and sEMG modelling [46]. Two established methods exist for electrode placement: longitudinally, along the long axis of the muscle, and transversely, across the long axis of the muscle [47]. For longitudinal placement, the bipolar electrode is placed halfway from the distal motor-end plate zone, which is close to the muscle mid-line and the distal tendon. This placement avoids the IZ and the sensor does not overlay the tendon while it is in motion. Bipolar electrodes that are placed transversely should be placed on the muscle, but avoiding the boundary of the muscle recording area. As a general rule, one should place the electrodes so that their mid-point is parallel to the long axis of the muscle.

Rainoldi *et al.* [48] studied the location of the IZ of 13 superficial muscles in the lower limb and the degree of inter-subject uniformity of the IZ in order to determine a standard and optimal electrode location (OEL) procedure. It was found that for some of the 13 muscle groups there was an amount of uniformity of the IZ if the signal had high quality. It was therefore concluded that OELs exist for some muscles in the lower extremity and a standard technique to locate the IZ was presented to ensure that EMG electrodes are placed between the IZ and the tendon. Nishihara *et al.* [49] had similar findings when identifying the OEL in fusiform and deltoid muscles. The former are more simple structures and signal propagation from these muscles is easily observed in the raw EMG signals, while the latter has a more complex muscle structure. The findings from that study revealed that the sEMG signals can easily and automatically estimate the signal propagation pattern and the optimal placement of the electrodes, even for the more complex deltoid muscles. In a recent IZ study, DeFrietas *et al.* [50] found that the IZ is different for men and women and that IZ in the biceps brachii location changes with movements in the elbow joint angle, although this was not related to weight, height, or humerus length. Another interesting point was that the IZ location can be more accurately estimated in relative (*i.e.*, percentage of the humerus length) rather than absolute (*i.e.*, distance from the acromion process) terms.

Accurate results rely heavily on accurate knowledge of the IZ location. Nielsen *et al.* [51] found that the distribution of the IZ substantially affects the muscle fibre conduction velocity (MFCV) estimated by sEMG signals. Farina *et al.* [52] argued that an accurate method for determining inter-electrode distance (IED) and electrode placement will result in reliable indications of muscle fatigue. In their study on the fatiguing upper trapezius muscle, they suggested that a distance of 20 mm between a single pair of electrodes is necessary in order to eliminate crosstalk and reduce the sensitivity of the EMG descriptors to noise and crosstalk. The optimal electrode location was determined based on set selection criteria that were the same for all arm positions, EMG descriptors and IEDs.

In experimental research there is often variability in electrode placement between subjects, unless each subject is individually and substantially tested. A study by Finni [53] specifically trained two personnel in electrode placement and found that even with training, they had the same level of variability in their placement of the electrodes. When the variations were calculated they did not differ from each other and they concluded that variability in lateral positioning of the electrodes cannot be avoided even with trained personnel, although one should aim to place the electrodes over the muscle mid-line. It has also been found that the IZ changes at different levels of isometric contractions, and the increasing force causes a shortening of the muscle fibres which results in a lengthening of the distal tendon [54]. Since electrode placement greatly influences the results of the sEMG signals, it is advisable to report on their placement in a study to facilitate comparison as different electrode placement over the same muscle will produce differences in the sEMG recordings [55,56].

### 3.4. Signal Noise

One of the limitations of EMG is the various sources of noise acquired with the signal. Electromagnetic radiation is produced by electrical circuits within the equipment as well as from the surfaces of all “hot bodies”, including human bodies and inanimate objects. The EMG electrodes themselves produce transducer noise when they are in contact with the skin. Chemical reactions may take place in the contact region between the electrodes and conductive gel causing differences in electrical impedance [43]. Motion arising for example between the electrode interface and electrode cable, may also cause noise by creating irregularities in the data; however, the design of the equipment and the experimental set-up can reduce this noise. Normally EMG has an amplitude range of 0–10 mV before amplification, however, the amplitude of the signal is naturally random. The firing rate of the motor units (MUs), which is normally in the frequency range 0–20 Hz [36], also affects the EMG signal, acting as a noise source, which should be suppressed. Whilst technology improvements have decreased the level of noise in EMG signal acquisition, it can never be eliminated and should not require unnecessary filtering or create signal peaks [36]. To assist in this process, the factors affecting EMG signal noise have been classified and suitable algorithms designed to minimise noise and optimise equipment for noise reduction. EMG signal quality can be improved by identifying the signal-to-noise ratio that maximises the level of information from the EMG signal whilst minimising distortion due to noise.

### 3.5. Other Factors Affecting Signal Quality

Physiological factors also influence the quality of the EMG signal, including the quantity of tissue between the electrode and the surface of the muscle, the number of active MUs, blood flow, fibre diameter, the depth and location of active fibres, fibre type composition, *etc.* These factors vary independently among different muscles in the body. Other factors that influence the EMG signal are [43]:

- the timing and intensity of muscle contraction,
- the properties of the overlying tissue,
- the properties of the electrode and amplifier,
- the electrical properties of the contact between the electrode and the skin,
- the distance of the electrodes for the active muscle area.
- the gel material used on the electrodes.

### 3.6. Application of EMG in Muscle Fatigue Research

EMG is an easy to use technique and has therefore been used in a vast range of research on muscle physiology. Generally, localised muscle fatigue occurs after a prolonged, relatively strong muscle activity, when a muscle or a group of muscles are fatigued. Due to the variability of inter-person muscle characteristics, there is no simple function of muscle load and timing that defines a precise muscle fatigue threshold. Surface electromyography (sEMG) signals provide valuable information about the changes in the muscle when measuring and analysing localised muscle fatigue [57–59]. Changes in the EMG signals caused by fatigue are either measured in the time or frequency domain. Integrated EMG (IEMG) usually uses the time domain, and an increase in the signal period, amplitude and power reflects a higher muscle fibre recruitment for a fixed external force. The changes in EMG signal in the frequency domain relate to the median power frequency (MPF), which varies due to a shift towards lower frequencies, a small increase in low-frequency signal power, a relative decrease in high-frequency signal power, a decrease in low-frequency spectrum slope or an increase in high-frequency spectrum slope [58,60–63]. There are several reasons for these changes in the EMG signal, such as signal synchronisation, modulation of the recruitment firing rate, grouping and slowing of the conduction velocity [60,64,65].

Although sEMG has been applied in many studies of localised muscle fatigue, it is not without its limitations, in particular, in studies of dynamic muscle contractions. The use of sEMG requires proper knowledge of the signal generation and propagation mechanism. Although signal acquisition *per se* is easy, inaccurate conclusions are easily drawn when inappropriate experimental methods are used [39].

Most research concentrates on isometric contractions to establish typical sEMG readings when conducted in controlled settings. Changes in sEMG amplitude and centre frequency have been studied by Petrofsky *et al.* [58], who found a decrease in the centre frequency of the spectrogram for all muscle groups. Research has also shown that a development in muscle fatigue correlates with changes in sEMG signal amplitude and MDF [57]. Muscle fatigue causes MU recruitment and the MU firing rate increases as a function of the elapsed time. However, these changes are not reflected in the EMG changes which occur during fatiguing isometric contraction of the arm flexors at 20%–30% MVC [66]. It was recently found that the changes due to fatigue in the sEMG signal (increased amplitude and decreased frequency) suggest that the recruitment of MU firing rates correlates with sEMG amplitude [67].

Although MFCV strongly influences the power density spectrum (PDS) and has the highest inter-person repeatability, it has been argued that fatigue also compresses the frequency content of the sEMG signal in a proportional manner [68]. The PSD time-dependency can also be analysed, and is usually estimated from the instantaneous sEMG parameters, although there are shortcomings in the identification of changes in the short-period sEMG signals. A time-varying autoregressive model (TVAR) was proposed by Zhang *et al.*, which produced a more stable and accurate instantaneous parameter estimation [69]. Minning *et al.* [70] studied differences in the rate of fatigue in the shoulder muscles during voluntary isometric contractions. They discovered day-to-day inconsistencies in the rate of fatigue in the middle deltoid muscle which was also fatiguing more rapidly than other muscle groups. However, for the other muscles they found a consistent relationship between trial, day and muscle type. In a study on the relationship between short-time Fourier transform (STFT) and continuous wavelet transforms to analyse EMG signals from the back and hip muscles during fatiguing isometric contractions, it was found that the two methods reveal similar information about EMG spectral variables [71].

Although the success of sEMG is likely to be more prevalent in isometric muscle contractions, more recently EMG has been applied to dynamic contractions [72]. The analysis of narrowing windows of sEMG spectrum during cycling activities reveals a strong correlation between the onset of fatigue and the reduction of the MDF in dynamic contractions. What’s more sEMG has been validated using biochemical analysis, indicating that the low-frequency band is a reliable indicator of muscle fatigue in dynamic contractions [73]. By analysing the quantitative and qualitative changes in EMG patterns, such as IEMG and the frequency of the mean power, it has been argued that in dynamic contractions fatigue is related to qualitative changes in the pattern of MU recruitment, which occurs at a faster rate when the muscle has a higher degree of fast twitch muscles fibres. For the quantitative changes, only a small reduction in the IEMG was related to a high percentage of slow twitch muscle fibres [74]. Masuda *et al.* [45] studied changes in sEMG patterns during static and dynamic fatiguing contractions by looking at the MFCV, MDF and mean amplitude in the vastus lateralis. The MFCV appeared to be influenced by the metabolic state in the muscle, as it significantly decreased in isometric contractions, while it remained constant during dynamic contractions. This suggests that changes in the MDF cannot be explained wholly by shifts in the MFCV. Farina *et al.* [75] proposed a technique for detection and processing of muscle conduction velocity (CV) during dynamic contractions; they showed that a decline in CV reflects muscle fatigue. Another method for estimating muscle fatigue during dynamic contraction is to use a source separation technique related to independent component analysis (ICA) to test whether the firing of MUs becomes more synchronised at the onset of localised muscle fatigue. As argued by Naik *et al.* [76], it is widely accepted that lower-frequency sEMG signals indicate muscle fatigue due to MU synchronisation; however, there is little experimental evidence of this theory. Naik *et al.* concluded that during cycling movements, a global matrix is an applicable measurement for estimating localised muscle fatigue.

Several studies have identified the state of peripheral fatigue [7,32,41,77]. In a recent study, Gonzalez-Izal *et al.* [41] compared several EMG parameters to assess peripheral fatigue during dynamic contractions. The new spectral indices (FInsm5) developed by Dimitrov *et al.* [77], which are based on discrete wavelet transforms, were compared to spectral parameters, such as mean average voltage, MDF and ratios between spectral moments. Results showed these newly proposed spectral indices to be the best for assessing peripheral fatigue, both in correlation with the power output changes and in their regression. These new spectral indices have also been shown to be a useful tool in detecting changes in muscle power output in fatiguing dynamic contractions, and they can be used as predictors of changes in muscle power output [78]

Detecting muscle fatigue in an automated system requires a real-time measurement of changes in localised muscle fatigue. As early as 1987, Kramer *et al.* [79] proposed a robust and relatively reliable parameter of fatigue that could be calculated off-line from computed, real-time sEMG data obtained from a simple analogue device. Wavelet coefficients can be used in non-stationary and time-varying signal processing, hence they have been applied in the assessment of localised muscle fatigue for both static and dynamic contractions using sEMG signals. The amplitude of approximation coefficients coincide with muscle fatigue development. Moshou *et al.* [80] proposed a method for automating the detection of muscle fatigue by using neural networks, where a two-dimensional self-organising map visualises the approximation of wavelet coefficients, enabling the visualisation of the onset of fatigue over time, and thus separating the EMG signal from fresh and fatigued muscles.

### 3.7. Discussion

Several misconceptions about sEMG exist, and it is important for the researcher to be aware of its sometimes non-obvious limitations [81]. The sEMG signal can be influenced by factors which are not related to the exercise being studied, such as biomechanical factors and movement speed [82,83]. In such cases changes in muscle activation are not necessarily reflected in the EMG signal amplitude. Although it is generally believed that activation from the central nervous system generates higher EMG amplitudes, this assumption was recently dismissed [84]. When studying muscle fatigue using sEMG, it is mainly the spectral frequencies of the signal that are analysed, which are computed by various methods, such as autoregressive models, Cohen’s class time-frequency distributions and wavelet analysis. Research has shown that Cohen’s class time-frequency distributions and wavelet analysis may be more appropriate to study the non-stationary signals during dynamic contractions [81]. Nevertheless, the spectral analysis of sEMG has critical and intrinsic limitations. In studies of muscle fatigue the relations between conduction velocity and spectral frequencies are investigated, which identify MU activity and recruitment. Although the changes in the spectral frequencies and CV are often shown to have a linear relationship, this relationship is questionable in contractions with a high number of MU changes [85]. sEMG analysis for muscle fatigue during dynamic contractions is complicated and requires caution as several factors such as change in the number of active MUs, changes in force/power though the range of motion, changes in fibre and muscle length, together with the change in MFCV due to muscle fatigue, influence the non-stationary nature of the EMG signal, causing it to increase [41,75,86–88].

Kollmitzer *et al.* [89] reported that in clinical applications the most suitable measurement is EMG for sub-maximal MVC of the rectus femoris muscle. In another study, Rudroff *et al.* [90] found that sEMG is better suited for the detection of alterations in the muscle activation of the fatiguing muscle during sub-maximal isometric contractions compared to ultrasound. As already noted, several studies on muscle fatigue using other techniques, such as NIRS and SMG, have used them in conjunction with sEMG, stating that these techniques serve as additional measures, giving more information about the fatiguing muscle. This suggests that sEMG is the most suited measurement for detection and prediction of muscle fatigue; hence, this paper will focus on sEMG signal analysis.

## 4. Other Signal Modalities Compared to sEMG

Generally clinical studies of muscle fatigue acquire signals using two main techniques, MMG and/or EMG. Historically, EMG has been chosen as the most suitable clinical research tool. MMG, on the other hand, is considered to be a mechanical equivalent of sEMG [91] by recording the low-frequency oscillations produced by the muscle fibres when the muscle expands. There are also several other sensors and signal acquisition methods which have been used in studies on muscle fatigue, which are described in Section 4.5 below.

### 4.1. Ultrasound (SMG)

Sonomyography (SMG) uses ultrasound to describe the structural and morphological changes of skeletal muscles. Shi *et al.* [92] recently used SMG to accurately detect the morphological changes in the fatiguing muscle and stated that SMG can be used as an additional method together with sEMG, providing more information about the fatiguing muscle. The synchronous use of SMG and EMG is possible and may open up new possibilities for the investigation of the muscle and muscle fatigue in both static and dynamic contractions. However, a systematic method to synchronise the two signals is necessary [93]. Huang *et al.* [93] proposed such a method for the real-time study of contractions of various muscles and muscle fatigue. There have been concerns that a combination of SMG and sEMG signals will cause inconsistencies in the recordings of the surface electrodes due to the use of ultrasound gel. However, a study by Huang *et al.*[93] concluded that sEMG signals are not significantly affected by such gel. Recent research by Zheng *et al.* [94] used SMG instead of EMG to study the potential of the dimensional change in the muscle and used SMG for musculoskeletal assessment and control, finding that SMG can be well employed in prosthetic control. Guo *et al.* [20] confirmed this by comparing SMG to EMG in forearm muscles on a guided wrist extension, with the results suggesting that the SMG signal has great potential as an alternative method to EMG for evaluating muscle function and prosthetic control, although EMG is a more established method.

### 4.2. NIRS

During exercise, the intramuscular pressure restricts the blood flow, causing a significant decrease in oxygenation and blood volume. On the other hand, muscle contraction demands more oxygen delivery to the area, which increases blood flow. Changes in blood flow near (< 1 inch deep) the skin can be detected using near-infrared spectroscopy (NIRS), a non-invasive method that uses the near infrared part of the electromagnetic spectrum to measure the absorption properties of blood haemoglobin [95]. Light is absorbed equally for both oxygenated and deoxygenated haemoglobin at 800 nm, while at 760 nm it is mainly absorbed by deoxygenated haemoglobin. In NIRS, the absorption data from these two wavelengths give an index of deoxygenation, providing information about the oxygen levels in the blood within the muscle. NIRS can be used as a measure of oxygenation changes in a fatiguing muscle [96], revealing information about local blood circulation, blood volume and changes in the oxygenated haemoglobin in the contracting muscle.

Yoshitake *et al.* [97] studied the aetiology of the lower back muscle by simultaneously recording MMG, EMG and NIRS from the centre of the erector spinae from subjects while they performed isometric back extensions for 60 s at an angle of 15° with reference to the horizontal plane. The NIRS recordings found that muscle BV and oxygenation decreased dramatically at the onset of the contraction, but were then static for the remainder of the contraction. This can be explained by the restricted blood flow in the contracting muscle caused by the high intramuscular pressure during isometric movement, suggesting that this is one of the most important factors causing muscle fatigue in the lower back muscles. These findings suggest that NIRS, together with EMG and MMG, may yield reliable information about muscle fatigue, in particular for the lower back muscles.

Praagman [98] used NIRS and EMG to determine local oxygen consumption in relation to external force in a study of isometric contractions of the elbow flexor up to 70% MVC. A linear relationship was found for both the local oxygen consumption and the sEMG moment in relation to the flexion moment, suggesting that NIRS can be used in research on musculoskeletal models.

A recent study by Taelman *et al.* [96] investigated the relationship between sEMG and NIRS parameters for both isometric static and semi-dynamic contractions of the biceps brachii, in order to obtain supplemental information on muscle fatigue. The sEMG parameters (MPF, RMS) revealed muscle fatigue to be a linear process, starting at the beginning of a contraction, confirming the results of previous studies [99]. NIRS did not demonstrate such a linear process, but rather measured the velocity of fatigue progression, where the tissue oxygenation index (TOI) showed a correlation with the duration of the contraction. This suggests a strong relationship between the frequency content of the sEMG and the TOI, similar to findings of other research [100]. These findings suggests that both NIRS and sEMG used together reveal complementary information about muscle fatigue.

Muthalib *et al.* [101] recently studied the reliability of using NIRS during sustained and repeated isometric contractions. Subjects performed 10 s sustained and 30 s repeated isometric contractions of the biceps brachii at 30% and 100% MVC. It was found that the TOI is a reliable NIRS parameter to determine biceps brachii oxygenation in both sustained and repeated isometric contractions.

In summary, it has been shown that NIRS can be used for the understanding of oxidative metabolism in healthy muscle [102], but future research should further investigate its application in the study of muscle fatigue.

### 4.3. MMG

MMG measures the mechanical signal which can be observed from the surface of a contracting muscle and recording the vibrations in the muscle as the muscle fibres move [103]. MMG has been applied in clinical research using wireless technology. It has been found that MMG is capable of detecting individual muscle actions and can distinguish between central and peripheral muscle fatigue [15,104]. In recent years, this technique has been employed in research of prosthetic control and assistive technology for the disabled [105]. MMG signals have been recorded using various sensors, such as hydrophones, condenser microphones, piezoelectric contact sensors, goniometer, accelerometer, and laser distance sensors [103,106].

Goniometer is a composite word derived from the Greek words gonia, meaning angle, and metron, meaning to measure. It therefore follows that a goniometer is a device used to measure the angle between two connected objects (e.g., the knee), making it ideal for use in physical and occupational therapy, where it measures changes in the angle measurements of the joints. The goniometer has been used in the study of localised muscle fatigue of both static and dynamic contractions [33,107]. An important consideration in selecting the most appropriate goniometer sensor is that it must be capable of reaching across the joint, so that the two end blocks can be mounted where least movement occurs between the skin and underlying skeletal structure. In most studies the use of the word goniometer actually refers to an “electro-goniometer”, *i.e.*, an electrical or electronic device for measuring joint angles. This usage applies throughout this review paper.

As already stated, the goniometer can be used as an indicator of localised muscle fatigue. When performing an endurance task, either static or dynamic, a drop in the joint angle to a set threshold indicates fatigue. Masuda *et al.* [45] determined when fatigue occurred in the knee at a 90° joint angle during static and dynamic contractions by combining parameters derived from the sEMG signals with goniometer and temperature data. Ravier *et al.* [108] used a goniometer to compare the changes in the elbow angle at 100° for static contractions in the biceps brachii to determine the shift in the median frequencies of the EMG signal when fatigue occurs. Although the goniometer can be used in isolation to determine muscle fatigue, it is often used in conjunction with other methods as a means of providing additional information. The goniometer has been used in combination with EMG, mechanomyography (MMG) and ultrasound in various muscle groups to measure localised muscle fatigue [16–22].

The goniometer has limitations if the range of motion is very small (*i.e.*, *<* 1.0°). Chabran *et al.* [109] studied postural muscle fatigue in various muscle groups and found that angular displacement of the shoulder joint was too low to be measured by a goniometer in some tasks and instead used sEMG together with acceleration of the angular displacement of the shoulder joint to determine occurrence of fatigue. Studies by Al Mulla *et al.* [32] used a fuzzy classifier on goniometer signals to aid in the classification of sEMG signals. The goniometer signal contained two important aspects when assessing isometric contractions, elbow angle and oscillation (*i.e.*, standard deviation of the elbow angle), which are both reliable indicators in healthy individuals when assessing muscle fatigue. The use of these variables defined the boundaries of the sEMG signal (Non-Fatigue, Transition-to-Fatigue and Fatigue), providing the basis for training the sEMG classifier.

An accelerometer measures acceleration forces, which can be both static or dynamic. When used for static contraction it measures the tilt angle of a test object, while it senses the vibration in dynamic contractions, measuring the way in which the test object is moving. The accelerometer has been applied in research on muscle fatigue as a means to detect changes in the exercises performed by subjects. Fallen *et al.* [110] used a triaxial accelerometer in conjunction with MMG to measure the oscillation in muscle during contractions in order to detect peripheral fatigue during neuromuscular electrical stimulation protocols. Using the three axes of the accelerometer, the variations were better captured, giving a more accurate representation of the oscillation. In a study on muscle fatigue with voluntary and evoked (creating muscle twitches by supramaximal percutaneous ulnar nerve stimulation) muscle vibrations, Barry [15] used an accelerometer to measure changes on vibration amplitude. A relation was found between the vibration amplitude from evoked muscle twitches and the evoked twitch force from fatiguing muscle, as well as a relation between the vibration from evoked muscle twitches and vibration measurements during voluntary contractions.

Microphones can be used to detect the pressure waves that are produced by contracting muscles. Since the pressure waves are of low energy content, a very sensitive microphone is required to detect them. A high sensitivity microphone creates more voltage than a standard microphone, resulting in less need for amplification, but increased noise sensitivity. Courteville *et al.* [111] developed a high-sensitivity human-vibration measurement sensor, which works like a microphone, using an acoustic impedance that measures skin surface vibrations in the range 2 Hz to 1 kHz, which can be calibrated for use in clinical research. Watakabe *et al.* [112] used a condenser microphone to study the mechanical variables indicated by its signals (acceleration, velocity or displacement) and found it to be suitable as a displacement meter in MMG studies of sustained and dynamic muscle contractions. The condenser microphone can also be used to acquire the MMG signal in cycling exercises in order to detect fatigue-related changes [113]. Jaskolska *et al.* [114] compared MMG signals recorded by an accelerometer and a condenser microphone for sub-maximal isometric, concentric and eccentric contractions in the biceps brachii. Although the accelerometer and microphone had similar recording properties, the information acquired was different for all the three types of contractions with increased intensity. However, they concluded that both an accelerometer and a condenser microphone can be applied in the study of concentric contractions with low intensity. Thus, high-sensitivity microphones reveal important information about the muscle and can be used as a tool in the detection of the onset of fatigue.

The MMG signal is produced by muscle activity in dynamic as well as in isometric contractions. Several studies have demonstrated that the MMG signal amplitude can be used in the recording of muscle fatigue during dynamic contractions as it provides information about the motor control strategies. However several factors can, in theory, affect its amplitude and frequency, such as the changes in torque production, muscle length and tissue thickness, making it more difficult to understand the motor control strategies [115], and often leading to the avoidance of the use of MMG in studies of localised muscle fatigue with dynamic contractions [116]. At the onset of a contraction, the MMG signal shows a large peak due to changes in the muscle shape. Initially, studies on MMG focused on isometric contractions as the stationary signal is believed to be easier to analyse.

In concentric and eccentric contractions, the MMG signal amplitude increases with power input [117–120] which is accompanied by a peak in torque production [121–123]. However, this increase is smaller in eccentric rather than concentric muscle activity, consistent with findings from EMG recordings [124,125]. Shinohara *et al.* compared MMG with sEMG by using MMG to study the quadriceps muscle during maximal incremental cycle ergometry with increased power output. The study provided convincing evidence that MMG reflects muscle activity during dynamic contractions, with a linear relationship between the power output and MMG signal amplitude and an MMG signal that had only a small but stable level of noise [118].

MMG has been applied in research on isometric or sustained contractions and several studies have found that MMG is a useful tool in monitoring muscle fatigue under such conditions [113,126]. Orizio *et al.* [106] used an accelerometer to measure surface oscillations of the tibialis anterior muscle to investigate changes in the MMG before and after fatigue. It was found that MMG characteristics correlate to specific aspects of muscle mechanics, demonstrating MMG as a suitable technique to record changes in the contracting muscle when studying localised muscle fatigue. In another study, Orizio *et al.* [103] studied the role of MU recruitment and firing rate to determine the characteristics of MMG while performing voluntary and stimulated contractions. These results were compared to short isometric contractions in fresh and fatigued biceps brachii acquired with both MMG and EMG signals. The experiment was designed to reveal whether changes in MU activation in voluntary contractions would give similar results to the time and frequency domains of the MMG. It was concluded that the RMS of the MMG signal did not present any changes and the median frequency (MDF) shifted towards lower values when the muscle became fatigued.

In a study on the relationship between the MMG signal and muscle force in the biceps brachii, it was found that MMG enables deeper insight into the MU activation mechanism during voluntary ramp contractions compared to separate contractions. In addition, the study concluded that ramp contractions describe the relationship between MMG and force at higher resolution, thus reducing the time needed for data acquisition and reducing the risk of fatigue [127]

In studies of localised muscle fatigue in dynamic contractions, the MMG amplitude has been investigated for both concentric [128,129] and eccentric muscle contractions [130]. The linear relationship between the MMG amplitude and the work load was confirmed for studies on rectus femoris and vastus lateralis at maximal concentric isokinetic leg extension at different velocities. However, in the vastus medialis muscle, MMG amplitude decreased quadratically with work load [128]. The decrease in MMG amplitude can been explained by “muscle wisdom”, where the fatiguing muscle is activated economically as the central nervous system reduces the MU firing to compensate for muscle fatigue, which in turn reduces the number of pressure waves recorded by MMG [24]. An alternative explanation is that the muscle elasticity is reduced due to intramuscular pressure, muscle thickness and fluid content increasing over time in both static and dynamic contractions, which influences the muscle oscillations and pressures waves recorded by the MMG [131–134]. In a recent study, Søgaard *et al.* [135] argued that intramuscular pressure does not have an affect on the MMG amplitude. After studying muscle actions from the biceps brachii at various MVC during isometric ramp contractions, they found that even when the intramuscular pressure was increased, the MMG amplitude showed a linear relationship with force. Nevertheless, more research is needed for various muscle groups and different torque levels to provide sufficient evidence to support this suggestion.

### 4.4. Acoustic Myography, AMG

Acoustic myography (AMG) is a special application of MMG, whereby the sounds produced by muscle contractions are recorded. As the force of contraction increases within the muscle, the level of the sound produced increases and can be recorded using a phonocardiograph [136]. In a study carried out in 1985, Barry [136] compared sEMG with sounds from the muscle in order to distinguish between the electrical and mechanical events that occur as MUs fatigue during rest, intermittent contractions and sustained contractions. It was shown that, contrary to the sEMG amplitude, the acoustic amplitude decreased with fatigue, while for decreased efforts both the acoustic amplitude and the amplitude of the sEMG declined simultaneously. In addition, comparison of acoustic signals with needle EMG suggested that acoustic signal analysis of muscles can be used as a non-invasive method for measuring MU fatigue and that this method may be useful in differentiating muscle fatigue from decreased volition. Rodriguez *et al.* [137] compared the RMS of acoustic signals to the RMS of EMG signals, MDF of EMG and quadriceps torque of fatiguing muscle performing isometric contractions. It was concluded that the RMS-AMG signal differs from the recording of the other signals both during fatiguing contractions and during recovery, suggesting that the RMS-AMG signal contains additional information. Stokes and Dalton [138] also used AMG to investigate sounds from the fatiguing quadriceps muscle during voluntary isometric contractions, using sEMG as a comparison where both signals were integrated, giving IEMG and IAMG. This study demonstrated AMG as a new technique for the non-invasive assessment of fatigue and force production, with potential use in the fields of neuromuscular physiology and rehabilitation.

AMG has also been applied in studies of dynamic contractions and Dalton and Stokes argued that AMG mirrors changes in the force during both dynamic concentric and eccentric contractions of the biceps brachii [121]. They studied AMG frequencies in both isometric contractions of fresh and fatigued muscle in the quadriceps and during dynamic contractions of the biceps brachii, analysing the MPF of the AMG signal, and found that for both muscles the AMG-MPF had low frequency range in the various types of contractions which did not change with fatigue. Although much of the research on acoustic myography is somewhat dated, the technique remains in use but tends to be referred to simply as MMG rather than as AMG in ongoing studies.

### 4.5. Other Signal Detection Methods Used in Muscle Fatigue Research

#### Force Gauge

A force gauge is a device that can mechanically or digitally measure the force of a pull or push test. A digital force gauge measures strain by detecting the voltage of a load cell, which is then processed to calculate a force value with the most common output reading being the peak force. In research on muscle fatigue, force gauges have been used to measure the strain or pull on the muscle with applications in sport science, ergonomics, and clinical research. Thrasher *et al.* [139] studied functional electric simulation of muscle contractions by measuring the isometric muscle force using a strain gauge. The load cell was used to measure isometric knee extension and isometric dorsiflexion moments. Morris *et al.* [140] studied muscle fatigue during endurance exercises and used a four strain-gauge bridge torque transducer to measure fatigue occurrence in the quadriceps muscle. They used the force gauge data to calculate the percentage of the initial force lost over a 180 second period. Wright *et al.* [141] used an isometric strain gauge oriented in the plane perpendicular to the hand to measure the abduction force of the thumb. They found that when muscle fatigue occurs there is an increase in the central blood pressure, which may cause a decline in the muscle performance.

#### Moore-Garg Strain Index and CR Borg-Scale

Moore and Garg [142] developed a strain index as a method to assess workplace risks related to musculoskeletal disorders of the distal upper extremities (hand, wrist, elbow). This index divides a job into several tasks which are then assessed and placed in six categories based on their risk. According to Moore [142], the prediction of the onset and magnitude of localised muscle fatigue can be determined by certain criteria measuring the recovery time of the muscle between contractions. The index can also be used to ensure correct results in studies on muscle fatigue during contractions, as it gives a visual presentation of the subject’s state during the trials. Bernard [143] adopted the Moore-Garg technique and applied it to an observational scale when performing a task, which can be seen in Table 2, based on the same principle as the 10 point category ratio (CR10) Borg Scale. However, the Borg Scale is more widely applied in research on muscle fatigue. The CR10 point Borg Scale is a simple method of rating perceived exertion and can be used by sports coaches to assess an athlete’s level of exercise intensity in training and competition [143]. The 10 point scale is as follows [144].

Kankaanpaa *et al.* [145] used the Borg scale for subjective estimates of fatigue while performing sub-maximal back muscle endurance tests. To correlate between EMG spectral changes and subjective assessment of muscle fatigue in the back muscles, Dedring *et al.* [146] used the Borg Scale to classify subject’s performance. They found that the subjective Borg Scale rating correlated with EMG and endurance time, suggesting there is a strong link between subjective and objective measures of muscle fatigue. Similar results where found by Oberg *et al.* [147] when studying subjective and objective evaluations of fatigue in the shoulder muscle, where evaluations of subjects using the Borg Scale were comparable to the root mean square (RMS) and mean power frequency (MPF) of the sEMG signals. Since the Borg Scale index is used to evaluate subjective measures of fatigue, it may be difficult to compare only these ratings between subjects, as own perceptions of fatigue vary greatly. However, an expert may be able to use the scale more objectively to measure fatigue by identifying the various stages of mechanical fatigue taking place.

#### Modified Moore-Garg Strain Index

The Moore-Garg Strain Index has been modified by Al-Mulla *et al.* [107,148], similar to the method developed by Bernard, by reducing its complexity to enable easier translation into button presses without loosing important muscle fatigue observation. In this study the absence of a button press indicated a state of Non-Fatigue, one button press indicated Transition to Fatigue, while two button presses indicates its progression and finally, three button presses indicated Fatigue. Table 4 show the modified index:

### 4.6. Discussion

MMG has contributed greatly to the understanding of muscle activity, although it is often used simultaneously with EMG. It is thought that MMG should not be used for the study of dynamic muscle contractions due to the variety of factors affecting the complex MMG signal. Furthermore, it has also been noted that MMG has shortcomings in its reliability for the repeated measurement of muscle fatigue. Al-Zahrani *et al.* [149] cautioned against using traditional parameters (RMS, MPF, MF) with MMG in assessing muscle fatigue due to variability in day-to-day recordings of fatigue by MMG measurement.

Several studies have compared MMG with EMG in order to determine when and for what types of research they are suitable. Søgaard *et al.* [135] studied long term muscle fatigue in the biceps brachii during intermittent contractions recorded by both MMG and EMG and found that the response was more profound in MMG, suggesting it as a valuable measure for the detection of impairment in the excitation-contraction phase. Perry *et al.* [150] found a difference between MMG and EMG responses during fatiguing dynamic contractions of the quadriceps muscles. The dissociations were most evident when the MMG amplitude tracked torque production; however, differences were also recorded in MMG amplitude response for different velocity tests and between the different muscles in the quadriceps (superficial muscles and quadriceps femoris), suggesting that the MMG signal is influenced by changes in the muscle fibre. Coburn *et al.* [123] reported no significant relationship between eccentric torque and EMG wavelet centre frequency nor MMG amplitude, while Cramer *et al.* [82] found a proportionate increase in torque with MMG amplitude and concluded that MMG is best suited for reflecting the power output and sEMG amplitude reflects torque. However, Cramer *et al.* [82] argue that the two signals should be recorded simultaneously, if possible, as the signals from both MMG and EMG are distinctive and can contribute valuable information about the mechanical and electrical aspects of muscle strength and power. In a study validating MMG for assessing masseter muscle fatigue, Ioi *et al.* [151] concluded that MMG analysis should be combined with EMG to better investigate the masseter muscle status. Madeleine *et al.* [152] also concluded that using EMG and MMG in conjunction would give complementary information about localised muscle fatigue at low-level contractions.

The issue of gender differences in both MMG and EMG signals is one of current research. Cramer *et al.* [153] failed to find any gender differences in MMG and EMG signals. However, McGregor *et al.* [154] found that control entropy declined during prolonged activity for males, but not for females, suggesting a gender difference. These differences were also present in some of the EMG recordings. Kawczynski [155] also found gender differences in the EMG signals when investigating MMG and EMG changes during and after fatiguing shoulder eccentric contractions, although no gender differences were recorded in the MMG signals. These studies suggest that more research is needed into gender differences in both MMG and EMG signals since gender differences in MMG amplitudes can be explained by gender differences in muscle mass and thickness of the tissue layer [156], while there is no such explanation for EMG signal differences.

There are several techniques for signal detection which are often used in conjunction with each other for the study of muscle fatigue and it may be difficult to determine which to use in a particular application. Most modern research uses one or more of the methods described here in conjunction, such as an accelerometer with sEMG electrodes. Usually, the aim of combining sensors with sEMG or other sensors is validation, labelling or improving the signal to noise ratio.

To date, no consensus has been reached upon the ideal sensor technology to use for MMG recordings [111,157,158]. The literature suggests that accelerometers are more appropriate than condenser microphones due to background noise. Less importantly, accelerometers are inexpensive and reliable devices whereas condenser microphones are more expensive and have a much larger frequency range (20–2,000 Hz) than that needed for accurately recording muscle vibrations (13–35 Hz) [104].

Compared to sEMG data collection, accelerometers are physically bulkier, more susceptible to noise from sudden movement and are significantly more expensive than sEMG electrodes. Limitations of NIRS are related to inconsistencies regarding muscle oxygenation during isometric exercise, suggesting that it is a less reliable method. NIRS sensors are also very sensitive to movement, which makes NIRS an unsuitable candidate technique in sports and other movement-rich scenarios. sEMG electrodes are an accepted tool in clinical muscle fatigue research; sEMG electrodes are used to detect dynamically the changes in the muscle as it fatigues, with the output signal indicating any fluctuations in the muscle performance. sEMG electrodes are sensitive to electromagnetic noise and the signal should thus be filtered to improve the signal-to-noise ratio. Another issue concerns electrode placement, as variations in placement result in signal inconsistencies and crosstalk from other muscles, especially in the forearm region. However several methodologies have been proposed to overcome these problems, such as a standardised electrode placement method (sEMG) for the Non-Invasive Assessment of Muscles, SENIAM [46] and the use of electrode arrays [31]. Nevertheless, sEMG electrodes are widely used in studies on muscle fatigue, for both dynamic and isometric contractions.

It is possible to use the goniometer sensor to measure the development of fatigue in a realistic scenario [33,107]. However, currently available goniometer sensors are expensive, have a short lifetime and must be handled with care. Electronic force gauges are also applicable in measuring fatigue but suffer from fragile construction, high cost and subject encumbrance in most scenarios [107]. The Moore-Garg strain index and the modified Borg Scale can be used for fatigue detection in terms of translating facial/body cues using video processing, but these techniques suffer from privacy issues and can be highly subjective.

## 5. sEMG Signal Analysis and Feature Characterisation

sEMG signals can be analysed to detect muscle fatigue by examining the changes in EMG measurements. Studies on sEMG show that an increase in EMG signal amplitude or shifts in the spectrogram are indicators of muscle fatigue in static contractions [83,159–163]. Studies carried out by Hagberg [57] have established that significant changes in the sEMG signal indicate muscle fatigue. Studies on muscle fatigue during isometric contraction have established typical sEMG readings when conducted in controlled settings. Changes in sEMG amplitude and centre frequency were studied by Petrofsky *et al.* [58], who found a decrease in the centre frequency of the spectrogram for all muscle groups. It has also been shown that a development in muscle fatigue correlates with changes in amplitude and MDF [57]. A variety of parameters have been used to investigate sEMG signals to determine muscle fatigue; however, it is common to study the signal in terms of its frequency at a certain time, in both the time and time-frequency domains.

### 5.1. Time Domain and Frequency Domain Analysis

Signals are acquired, and in some circumstances, analysed, in the time domain where the signal amplitude/voltage is represented as a function of time. However, for many analysis techniques, it is the frequency of the signal that is of greater value, and consequently the signal should be analysed in the frequency-domain, whereby the signal undergoes a Fourier transform so that it is represented as a function of frequency rather than time.

Both the average rectified value (ARV), which measures the average of the absolute signal value, and the RMS, which is a measure of the signal power [1], are used in the analysis of the raw EMG signal in the time domain. The RMS of the EMG signal calculates the square root of the average power of the raw EMG signal over a specific time period [35]. De Luca’s research group [35] acknowledged both the ARV and RMS as appropriate analysis methods, however, several authors prefer the RMS [39,83], since it can be used to obtain a moving average. The moving average approach is used for processing raw EMG signals from dynamic contractions, as it identifies the rapid changes in the muscle activity during such contractions by using short duration sampling windows [164]. Merletti *et al.* [39] suggested that EMG analysis of dynamic contractions can make use of another processing method, the ‘linear envelope’, which uses a low pass filter to smooth the rectified EMG.

When a signal crosses the zero amplitude line, it is said to have made a “zero-crossing”. When applied to sEMG data, the general idea is that an active muscle will produce more action potential, and hence generate more zero crossings. However, at the onset of fatigue, the zero crossing rate drops dramatically due to the reduced conduction of electrical current in the muscle. Therefore zero-crossings are counted using geometric calculations to give an indication of the muscle status.

The total band power of the sEMG signal can be estimated using Welch’s method. This method has been used in several sEMG fatigue analysis studies and has proved to be useful in quantifying the power of the EMG signals [165].

The frequency content of a signal can be determined by performing a Fourier transform to reveal its individual frequency components. The fast Fourier transform (FFT), a method for calculating the discrete Fourier transform, is suitable for use in stationary signals. EMG signals, which are non-stationary, should be represented in both the time and frequency domains. Therefore, the short time Fourier transform (STFT), which analyses a small temporal section of the signal, can be used to determine the frequency and phase evolution of the EMG signal over time.

The time and frequency resolution depend upon the sampling rate and the temporal length of the signal section. Due to the inverse relationship between time and frequency in the Fourier transform, it follows that the higher the time resolution the lower the frequency resolution will be and vice versa [3]. The spectrogram of the signal is the squared magnitude of the STFT.

Dimitrov [77] proposed a new spectral parameter with higher sensitivity than traditional indices for both dynamic and isometric contractions, which is a valid and reliable tool for the assessment of muscle fatigue. Following an FFT, this parameter represents the ratio between the low- and high-order spectral moments of the EMG power spectrum. Gonzalez-Izal *et al.* [41] used this index to measure the changes in muscle power during a high-intensity dynamic protocol and compared it to other frequency and amplitude parameters. It was found that the logarithm of this index detects the changes most accurately by assessing peripheral impairments.

EMG signals can be analysed in the time-domain using the PDS to describe how the power of a signal is distributed among its frequency components. Significant changes in the power spectrum indicate muscle fatigue [166], such that after fatigue onset the PSD is increased in the low frequency components and decreased in the higher frequency components.

Two of the most common frequency-dependent features in sEMG analysis are the mean frequency (MNF) and MDF. The MNF is “the average frequency of the power spectrum and is defined as its first-order moment” [4], while the MDF is an index used in studies of spectral shifts and can be defined as “the frequency which divides the power spectrum in two parts with equal areas” [1]. The power spectrum represents the median frequency of the power, based on a continuous spectrum distribution. Hagberg stated that if the MDF decreases along with an increase in sEMGsignal amplitude, it is a strong indication of fatigue [57]. The spectral frequency can be redefined to represent the non-stationary nature of the signal, or the instantaneous frequency of the frequency content of the signal [88]. The instantaneous median frequency (IMF) was introduced by Roy *et al.* [165]:

(1) $$\int_{0}^{\mathrm{IMDF}(t)}P(t,w)\mathrm{dw}=\int_{\mathrm{IMDF}(t)}^{\infty }P(t,w)\mathrm{dw}$$

where *t* is time, *P* is the PSD function, *w* is the size of the temporal window and *d* is the depth of the signal.

Studies by Oskoei *et al.* concluded that a significant decline in the IMF of the signal is a significant manifestation of fatigue occurrence [4]. In addition, Georgakis *et al.* demonstrated that the average instantaneous frequency is superior to the mean and median frequencies for the analysis of muscle fatigue during sustained contractions [167].

Some analysis methodologies use both the time and frequency domains to analyse the EMG signal. For example, the Cohen class transformation, a time-frequency representation applied in biomedical signal processing, is well-suited for analysis of signals from dynamic contractions [168]. It is a distribution function introduced by Cohen in 1966 using bilinear transformations, giving clearer results than the STFT. However, due to its use of bilinear transformations, the Cohen class is affected by cross-term contamination in its analysis of several functions, which can be avoided using window functions. The Wigner-Ville distribution function (WVD), proposed by Wigner in 1936 [36], was first used for corrections to classical statistical mechanics, however, it is also applicable as a transform in time-frequency analysis. This transform has higher clarity than the STFT and has more properties than most other time-frequency transforms, using all available information in the EMG signal. In 1948 Ville revised this function into a quadratic representation of the local time-frequency energy of a signal. It was discovered by Ricamato *et al.* [169] that the WVD would detect the frequency ranges of the MUs, displaying recruitment patterns as muscles contract. However, Davies and Reisman [170] found that the WVD joint density spectrum is noisy although its localisation properties are excellent and “generally concentrated around the instantaneous frequency of the signal”. Another member of the Cohen’s class functions is the Choi-Williams distribution [170]; it makes use of kernels to reduce the interference, which the Cohen’s class distribution suffers from, although it is only possible for the kernel function to filter out the cross-term contamination.

There are many time-frequency functions which can be used to analyse sEMG signals during localised muscle fatigue. Studies by Davies and Reismann show that STFT can most precisely represent spectrum compression during muscle fatigue. Due to the cross-term contamination in the WVD, it is not possible to display the changes in the frequency components with muscle fatigue accurately. In a comparison between the STFT, the WVD, the continuous wavelet transform (CWT) and the Choi-Williams distribution, Karlsson *et al.* [171] found that the CWT resulted in a more precise estimation of EMG signals when applying various time-scale methods to analyse sEMG signals.

### 5.2. Wavelet Analysis

By using a wavelet function (WF), the wavelet transform (WT) decomposes a signal into numerous multi-resolution components [172,173]. It is used to detect and characterise the short time component within a non-stationary signal, providing information regarding the signal’s time-frequency. The WF, being both dilated and translated in time and a linear function that does not suffer from cross-terms, undertakes a two-dimensional cross correlation with the time domain sEMG signal, making it an excellent alternative to other time-frequency parameters [173].

There are a number of so-called “mother wavelets” that can be used for signal decomposition including Symm-let, Coiflet, Haar, Morlet, Daubechies and Mexican Hat [174]. To select the most appropriate mother wavelet for a specific application and signal type, the properties of the wavelet function and the characteristic of the signal should to be analysed and matched. Certain wavelets have somewhat established guidelines for their use, e.g., Db4 is said to be suited for signals using feature extractions and linear approximation with more than four samples, while Db6 is suited for signals that are approximated by a quadratic function over the support of six and finally coiflet6 is better suited for data compression results [175].

Guglielminotti and Merletti [176] hypothesised that if the wavelet analysis is selected to fit with the shape of the motor unit action potential (MUAP), the WT would give the best energy location in a time-scale. Kumar *et al.* [174] stated that the STFT does not give an optimal time or frequency resolution for the non-stationary signal, although the relatively short time windows may trace spectral variations with time. The WT, comprised of numerous WFs, can be used to decompose the sEMG signal. The output of the power transform domain is calculated and thus functions as a deciding parameter in selecting the most appropriate WF to give the highest contrast between sEMG cases. It has been shown that it is possible to detect muscle fatigue status by determining the Sym4 or Sym5 WFs and decomposing the signal at levels 8 and 9 (out of 10 levels). Kumar *et al.* [174] discussed the effectiveness of decomposing the EMG signal to measure its power in order to identify muscle fatigue as an automated process. Another study by Al Mulla *et al.* [177] used a genetic algorithm (GA) to create a pseudo-WF and an optimal decomposition scale was selected that specifically improved the classification of localised muscle fatigue using sEMG. The fine tuned pseudo-WF improved the classification of muscle fatigue significantly when compared to other standard WFs.

### 5.3. Regression Analysis

Regression statistics is used to determine the relationship between an independent variable or variables and a dependent variable. An autoregressive (AR) model is a random process used in statistics and signal processing to model and predict natural phenomena. In a study by Graupe and Cline [178] in 1975, the AR moving average (ARMA) model was developed to represent EMG signals, where the signals were split into short time intervals and the signal was considered to be stationary. However, this model was replaced in 1980 by Sherif’s model to be used on the non-stationary EMG signals, using the AR integrated moving average (ARIMA) model [179]. Hefftner *et al.* [180] exploited the computational speed of the AR model for EMG feature discrimination.

Kim *et al.* [181] measured fatigue in the trunk muscle using the first AR model, and concluded that the model was capable of assessing fatigue in static exercises, being sufficiently sensitive to detect fatigue at low force levels. Several authors have revised the AR parameters, adding a non-linear element (ARMA) [182] and a non-stationary identifier [183]. However, the ARIMA model is complex with a high computational cost and Tohru [184] argued that more accurate models (ARMA and ARIMA) are not needed for studies on dynamic contractions.

### 5.4. Composite Features

The term “composite features” relates to the use of a combination of common features to develop a new feature that aids in the analysis of sEMG signals. MacIsaac *et al.* [185] presented a mapping function that maps segments of multiple myoelectrical signal for fatigue estimation of dynamic contractions, where the inputs are time domain features. This function is tuned by artificial neural networks (ANN), and is capable of use in real time applications. Results show that this function better maps the sEMG signals than both mean frequency and instantaneous men frequency for different conditions.

A one-dimensional spectrogram (1D Spectro), which is a composite feature, was developed to assist in the prediction and detection of muscle fatigue, in particular the onset of Transition-To-Fatigue [33,34], being well-suited for non-isometric contraction analysis. It is thought that when both the instantaneous MDF and the total band power are unified by subtraction, they produce a feature that imitates a spectrogram and simplify it to its one-dimensional (1D) form. Therefore the standard deviation of this unified signal can be used to produce a feature 1D Spectro-std. Classification results using the 1D Spectro and 1D Spectro-std features outperform many commonly used features [33].

### 5.5. Fractal Indicators

A fractal is a geometric shape that splits, making smaller copies of the original; the fern leaf is a well-known example from the natural environment. Fractals have been demonstrated as a measure of EMG signal characteristics [186], where fractal indicators observe the chaotic EMG signal at different time scales, replacing it with a family of time scales [187]. Fractal indicators are sensitive to the force levels in isometric contractions, and have proved to be good measures of muscle fatigue [108,188]. Gang *et al.* [189] used a multi-fractal analysis to study the sEMG signals during static contractions. They found that the spectrum area increases with fatigue and hence can be an indicator of fatigue; this method had higher sensitivity compared to the MDF.

### 5.6. Entropy

Sung *et al.* [187] argue that entropic measures reveal part of the sEMG signals that are not included in the power spectrum and it can be a useful tool in detecting muscle fatigue in gender differences.

### 5.7. Recurrence Quantification Analysis

Recurrence quantification analysis, a method of nonlinear data analysis which is used for the investigation of dynamical systems, is highly effective in detecting changes in the sEMG signal and is almost equivalent to the frequency domain analysis of the signal in non-isometric contractions [190]. Morana *et al.* [191] recently used recurrence quantification analysis in a study of muscle fatigue and stated that this method can detect peripheral muscle fatigue.

### 5.8. Higher-Order Statistics

Higher-order statistics (HOS), a technique based on probability theory, characterises and analyses the nature of a random process, making it appropriate for use in the random time series produced by EMG signals. HOS has been used in sEMG studies to estimate the amplitude and the number of new MUAPs, as proposed by Kanosue *et al.* [192]. Several authors have studied HOS in sEMG processing, in particular testing it for Gaussianity, linearity, coherence and coupling of the signal. Their findings showed that during contractions at low and high force are non-Gaussian, while during the mid-level force the distribution is maximally Gaussian [36,193,194].

Blind source separation (BBS), a method based on neural networks that divides a linear mixture of statistical moments on a learning algorithm, was first suggested by Belouchrani *et al.* [195]. Farina *et al.* [196] found a new method, using spatial time-frequency distributions, to be able to distinguish between simulated and non-stationary sEMG signals, avoiding the problem of overlap of EMG signals from various muscles and thus avoiding the need for linear filtering with BBS. The limitations of BBS are generally not considered when applied in EMG signal processing. ICA is a variant of BBS and its first application to biomedical time series analysis was presented by Makeig *et al.* ICA aims to recover independent sources based on a mixture of linear functions that are not known in order to obtain a highly independent signal. Subasi *et al.* [197] used ANN with ICA to differentiate between signals from fresh and fatigued muscles, enabling visualisation of the onset of fatigue over time. A similar approach was applied by Naik *et al.* [76] where source dependent properties (such as ICA) were used to test the synchronisation of the firing unit at the onset of localised muscle fatigue during cycling movements.

### 5.9. Discussion

Feature extraction is perhaps the most important stage of data analysis in clinical applications since it impacts directly the performance of the developed application. Many features can be extracted relating to localised muscle fatigue. Some of the features use the frequency domain while others use the time domain or both. Other feature extraction techniques use fractals or machine learning. Wavelets can be used to reduce the signal to noise ratio whilst retaining the fatigue content. Feature extraction techniques that create composite features from other muscle fatigue features have been employed with a high degree of success, but this remains a little researched area in sEMG. In a study by Al Mulla *et al.* [34] the symmetry of both the frequency and the power features of the sEMG signal was used to derive a composite feature that is more sensitive also pinpointing the onset of Transition-To-Fatigue (peripheral fatigue). In addition, Al Mulla *et al.* [107] used a GA that used most of the features mentioned in this section. They concluded that composite features produce better classification then single features. Most of the features mentioned in this section produce exceptional classification results, however one must always consider which is the best feature to use for detecting or predicting localised muscle fatigue depending on the mode of application.

## 6. Feature Selection

In machine learning and statistics, as well as pattern recognition and data mining, feature selection is a technique whereby a subset of relevant features from the data is selected, which will be applied in a learning algorithm [198]. The optimal subset contains a low number of dimensions that will ensure accuracy and the remaining, irrelevant, dimensions are discarded. Feature selection typically creates a model that facilitates the generalisation of the unseen dimensions and may substantially enhance the comprehension of the classifier model which is produced [199]. In supervised learning, which has been thoroughly investigated, the aim is to select a feature subset which is producing high classification accuracy [199]. However, for unsupervised learning the goal is to identify an optimal subset that produces high quality clusters for a set number of clusters.

In research on localised muscle fatigue, feature selection is used to facilitate the pattern recognition and classification of the features analysing the sEMG signals [200,201]. Various methods have been applied in this process, however, the Davies Bouldin index for measuring clustering selection is commonly used for EMG pattern recognition [202–204]

### 6.1. Clustering and Class Separation

Clustering is generally considered as an unsupervised algorithm for clustering a heterogeneous population into a set of homogeneous group of classes. This strategy does not ensure grouping similar classes together. Al-Harbi and Rayward-Smith modified the k-means clustering algorithm to act as a classifier algorithm, which are able to group known classes using the k-means (Supervised k-means) [205]. This technique was used by Al Mulla *et al.* [7] to visualise the three different fatigue classes (Non-Fatigue, Transition-to-Fatigue and Fatigue) and to produce an index for measuring class separability.

#### Davis Bouldin Index

The Davis Bouldin index (DBI) is a metric evaluating clustering algorithm. It serves as a measure of cluster quality by calculating the distance of the cluster members to the cluster centroids and the distances between the cluster centroids. As such the DBI is a measure of the standard deviation of the signal [206,207]. The DBI can be expressed as follows:

(2) $$\mathrm{DBI}=\frac{\sum_{i=1}^{k}\mathrm{std}\left[\mathrm{dis}\left(C_{i},d^{0}\right),\cdots ,\mathrm{dis}\left(C_{i},d^{n}\right)\right]}{\mathrm{dis}\left(C_{0},C_{1},\cdots ,C_{k}\right)}$$

where *C_i_* is the centroid of the *i^th^* cluster and *d* the *n^th^* data member that belongs to the *i^th^* cluster. The Euclidian distance between *d^n^* and *C_i_* expressed by the function is *dis*(*d^n^*, *C_i_*). Furthermore, *k* is the total number of clusters and the standard deviation is *std*(). A small DBI indicates well separated and grouped clusters, which means that the lower the DBI the more separable are the classes.

There are several methods to measure cluster quality, but the DBI has been extensively applied in research on muscle fatigue [7,208]. The DB index is related to the performance of the linear Fisher discriminant classifier to pairwise clusters.

## 7. Classification

There are numerous ways to classify the sEMG signals, although the non-stationary nature of the signals make classification more complicated [209]. A number of classification methods used for sEMG fatigue related signals are described below.

One common method for sEMG classification is to measure the Euclidean distance between the MUAPs waveform, where a shimmer is generated in the representation of time-triggered and non-overlapping MUAPs [36]. The shimmer is influenced by external factors, such as background noise and noise from offsets. In addition, the shimmer of the MUAP is affected by the variance within a class as well as the distance between the classes.

Christodoulou and Pattichis [210] suggested using an ANN as a classification method, which can be implemented in three phases. The first phase is that of unsupervised learning, which is built on competitive learning and on a one-dimensional self-organising feature map. In the second phase the learning vector is quantified, this is a self-supervised learning method which aids classification performance. Finally, the third phase is that of classification. The fuzzy approach has been compared with the ANN method on four subjects and very similar classification results were obtained. It is superior to the latter in at least three points: slightly higher recognition rate, insensitivity to over-training, and consistent outputs demonstrating higher reliability [211].

### 7.1. Machine Learning

Machine learning is a method of developing algorithms which use empirical data (from sensors or databases) to allow computers to evolve programmes or behaviours themselves. Machine learning is closely related to artificial intelligence and pattern recognition. There are several approaches in machine learning but for EMG signal processing it has been suggested that neural networks provide a good solution. More specifically, the dynamic recurrent neural network, which has two different adaptive parameters using fully interconnected neuron-like units and which maps the relationship between arm movement and EMG muscle activity, was proposed by Cheron *et al.* in 1996 [212].

ANNs are non-linear statistical data modelling tools that are inspired by the structure of biological neural networks, and that are able to solve many problems that defeat other statistical methods. Del and Park [213] suggested ANNs as a suitable technique for real-time applications of EMG in 1994. Their method can precisely identify the EMG signals, and the EMG features are extracted by Fourier analysis, using a fuzzy algorithm for clustering. The operations are undertaken in real-time by a FFT performed by the multipliers in a digital signal processor. In a recent study, a newly developed evolved feature aimed at predicting the time to muscle fatigue used a supervised ANN that was a simple linear algorithm composed of five training inputs and one testing signal unseen by the ANN [148]. The rate of change of the five inputs was calculated using the first 20% of the evolved feature signal, then simplified to enable faster ANN training. The ANN adjusted its training weights by using time to fatigue for the five training signals, enabling it to predict the time to fatigue by using only 20% of the total sEMG signal with an average prediction error of 9.22%.

Fuzzy logic, a form of logic tolerant to contradictory data, is used in biomedical signal processing and classification to overcome problems where signals are stochastic and therefore may be contradictory in nature [211]. Fuzzy systems can be trained to identify patterns which are not identifiable by other methods. Fuzzy systems determine fuzzy operators on fuzzy sets, which may be unknown, requiring the use of ‘IF-THEN’ rules. Fuzzy systems are used to model or classify problems with variables and rules that can be analysed by a human user. A fuzzy classifier is an algorithm that labels objects by class and it is argued that the classifier can predict the class label. Kucheva *et al.* [214] argued that any classifier that uses fuzzy logic in its training set is a fuzzy classifier. A fuzzy system has a vector that contains the values of the features for a specific task. The system will run a training algorithm on a training data set. Once the system is trained it can be applied to unseen objects. There are several models of fuzzy classifiers, and the simplest method is the rule-based approach that works as an “IF-THEN” system, where the class label is the consequent part of the rule. If the consequent part of the rule contains linguistic values the output will be soft label with values from the discriminant function. Takagi and Sugeno [215] identified a fuzzy classifier where the function is the consequent. This method also works according to the if-then rule, however, the rule is a regressor over the feature data space.

**Figure 3.**
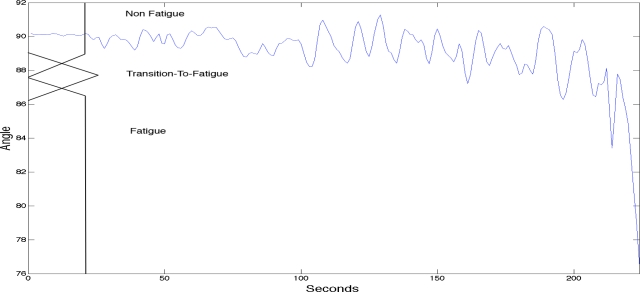
The use of elbow angle to label and classify the signal.

**Figure 4.**
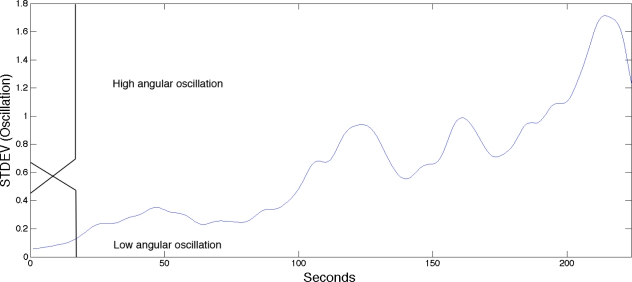
The use of angular oscillation to label and classify the signal.

Al-Mulla *et al.* [7,32,33] used a fuzzy classifier for setting the boundaries when labelling the sEMG signals for classification of localised muscle fatigue. The fuzzy classifier automated the classification process, although a human expert verified the outcome. For muscle fatigue during isometric contraction two main criteria were used using fuzzy logic terms. The two inputs were elbow angle and its standard deviation, and a single output see Table 5 for the rule base. Figure 3 indicates the fuzzy set input for the elbow angle provided by the goniometer (0 to 180 degrees). The figure also has a superimposed illustration of a single goniometer trial signal giving an example of how the fuzzy classifier is finding the boundaries to enable the labelling of the sEMG signal. Figure 4 indicates the fuzzy set input for the arm oscillations (*i.e.*, the standard deviation of the elbow angle), which was also provided by the goniometer: An increase in the standard deviation of the goniometer signals indicates either low angular oscillation or high angular oscillation. The increase in oscillations in particular is indeed a precursor of physiological fatigue.

Genetic Programming (GP), a specialisation within the field of Genetic Algorithms (GAs) [216] and based on Darwin’s theory of evolution, finds the best suited computer program to perform a set task. While GAs search the space of a function to find an optimum solution, GP creates computer programs as part of the solution. GP has proved to be a useful method to solve linear and nonlinear problems, where the areas of the state space are explored through mutation, crossover and selection operations applied to individuals in the population [217]. Raikova and Aladjov used hierarchical genetic algorithms (HGA) to investigate the motor control for muscle forces during dynamic conditions [218]. The HGA used genetic operators to find the moments of neural stimulation of all the MUs, which are the variables in genetic terms, so that the sum of MUs twitches fulfils the set goals. Results showed that HGAs are a well suited method to examine motor control. Al-Mulla *et al.* [32] used GP to classify localised muscle fatigue into three classes: Non-Fatigue, Transition-to-Fatigue and Non-Fatigue. The GP firstly labels the classes based on a fuzzy classifier and then is able to classify any unseen isometric sEMG signals. Al-Mulla *et al.* found that the GP classification is able to identify a Transition-to-Fatigue state, meaning that the GP can give a prediction of an oncoming fatigue. In a similar study by Kattan *et al.* [219], GP was used to detect localised muscle fatigue and the system was able to notify the user of possible approaching fatigue once it detects a Transition-to-Fatigue.

### 7.2. Linear Discriminant Analysis

In order to compare and validate features, a linear discriminant analysis (LDA) can be used. The following linear transformation describes the classification where the LDA maps the data (feature vector) y:

(3) $$y=w^{t}+w_{0}$$

where w and w0 are determined by maximising the ratio of between-class variance to within-class variance to guarantee maximal separability

### 7.3. Support Vector Machine (SVM)

SVM is essentially a supervised learning method for data analysis and pattern recognition, which can be used in classification and regression analysis. Its foundation is a concept of decision planes that define decision boundaries. The decision plane separates sets of objects with different class memberships. By undergoing training, the SVM uses an algorithm to develop a model that will predict which category the examples in the training set belongs to. SVMs are a useful technique of data classification [220,221].

### 7.4. One Clause at a Time, OCAT

OCAT is a classification function developed by Torvik *et al.* [222] in 1999, where the aim was to create a flexible but simple prediction function. In their study on predicting if a muscle is fatigued or rested by investigating the peaks and characteristics fractile frequencies in the EMG signals, they found that OCAT achieved the highest accuracy in comparison to other classification methods. Although ANNs also shows great accuracy, this classification method needs subjective fine tuning and is complex in its interpretations. Nevertheless, they acknowledged that the more classical methods might be more powerful as long as valid assumptions are made, which is why they stated that more research is needed. This is an interesting but fairly dated approach that attempts to predict localised muscle fatigue.

### 7.5. Discussion

Above is a description of various methods of classification for signal processing. All of these methods, either on their own or several at the time, have been applied in research on localised muscle fatigue. Some of the methods are well established in this field, such as machine learning including LDA and support vector machine. OCAT is a method that has received very little attention, although its classification has high accuracy compared to traditional classification methods. Another interesting point is that this method is trying to predict localised muscle fatigue, which might suggest that this method may be applicable in an autonomous system for the prediction of localised muscle fatigue, but more research is needed. GP is another newly applied method for the classification of localised muscle fatigue. In recent research, GP has been utlised for labelling and classifying sEMG signals from localised muscle fatigue [32]. In addition to producing high classification accuracy the system was also able to notify the user as fatigue was approaching. This finding is an important asset to the development of an autonomous system for fatigue detection and prediction.

## 8. Fields of Application for the Discussed Techniques

In this paper various techniques of studying localised muscle fatigue have been represented. The detection and classification of muscle fatigue adds important information to the fields of human-computer interactions, sport injuries and performance, ergonomics and prosthetics. An automated system that will predict and detect when fatigue occurs is especially useful in sports related scenarios, where fatigue can lead to injury. An automated system will guide the user in his training and act as a warning device before fatigue sets in to avoid unnecessary strain on the muscle promoting improvement and to prevent injury. This system can also be applied in occupational health and ergonomics, in particular where there is a risk of work-related musculoskeletal disorders. Localised muscle fatigue in the work place may cause injury, for example, if a task causes elevated static muscle activity [5]. An automated system can predict when the muscle is fatiguing and hence avoid injury in this scenario. Similarly, in ergonomics, a system like this can aid in the correction of posture problems before the occurrence of muscle strain or injuries.

The two main signal acquisition methods in studies on localised muscle fatigue are, as mentioned earlier, MMG and sEMG. These techniques have been used in various research and have been applied in different fields, in particular clinical research.

sEMG has been used for studies in human-machine interaction and physiology. The EMG signal can be used to detect medical conditions and to understand the nature of a muscle. For example, in the field of human-machine interaction, EMG signals from the forearm can be used as a control signal for a prosthetic device [223]. In addition to medical and physiological applications, EMG has been used by NASA as a control for flight systems, to develop an unvoiced speech recognition system where the EMG signal observes the activity of the muscles related to speech, and as a control signal for computer or devices where the EMG signal is an index for controlling a moving object [223].

Although MMG has mainly been applied in research on localised muscle fatigue during isometric contractions, Beck *et al.* [115] argue that MMG has usability for different during postural control following fatigue, making it applicable to fields such as ergonomics and occupational therapy. Applying this technique with wireless technology enables clinical examinations of daily activities in realtime settings [104]. MMG is a technique which is applicable across populations (both athletes and patients), and has been used for diagnosis and monitoring of neuromuscular disorders [127], as well as in gait and balance studies where the information is not obtainable using other traditional methods [104].

## 9. Concluding Discussion

This paper has described various methods in the study of localised muscle fatigue. It started with an explanation of muscle fatigue and the various muscle fatigue stages and then followed on to a representation of the various sensors that are used in order to detect fatigue. Further on, signal detection was discussed with a focus on MMG and EMG. Finally, the paper discussed feature extraction and classification methods that are used for the analysis of the sEMG signals.

A clear and widely adopted definition of fatigue is necessary to ensure that researchers have comparable results; however, to date, most research has only identified the Non-fatigue and Fatigue stages of localised muscle fatigue. Another aspect of muscle fatigue research has identified central and peripheral mechanisms of fatigue, suggesting that fatigue is not a single state, but rather contains different fatigue causing elements. Therefore, Al-Mulla *et al.* have identified the stage called Transition-to-Fatigue, which is the fatiguing state before complete exhaustion occurs. It is still not fully understood what happens during both central and peripheral fatigue, although some studies have detected some of the core difference between the two [13,24,30,31]. The Transition-to-Fatigue class is an important discovery for the real-time detection and prediction of fatigue, in particular for the development of an autonomous prediction/detection system. An interesting way to further current research would be to investigate if the Transition-to-Fatigue stage contains only peripheral fatigue or whether there are also elements of central fatigue present.

Various sensors are capable of providing different measures that aid in the detection of muscle fatigue, such as the angle of the joint, oscillations of the muscles, ultrasound images of the muscles and myoelectric signals emanating from the muscle. It may be difficult to decide which sensors should be chosen for a specific study and they are therefore often used in conjunction to improve the signal-to-noise ratio and to validate findings. To date, a consensus has not been reached on which is the best suited sensor for the detection of localised muscle fatigue. In MMG recordings, authors have favoured the accelerometer due to its reliability and low costs [104]. However, in comparison to sEMG electrodes, the accelerometer is more costly and is more easily influenced by noise. sEMG electrodes have been used widely in the study of localised muscle fatigue for both isometric and dynamic contractions, although this method has some shortcomings. Electrode placement is an area of concern to ensure reliability and repeatability of results. The IZ should be avoided and SENIAM has made several suggestions on how to place the electrodes. Nevertheless, electrodes are still considered as on of the more reliable sensors for the detection of localised muscle fatigue.

MMG and EMG are the two principal methods of signal acquisition used in the study of localised muscle fatigue. Other methods have been used, such as NIRS and SMG, however, these are mainly used to obtain additional information about the MMG and EMG signal. Studies on muscle fatigue using MMG have contributed to an increased understanding of muscle activity as well as the measurement of muscle fatigue. Nevertheless, the literature cautions against using MMG in the study of dynamic contraction, as well as for analysing signal with traditional parameters [149]. Thus, MMG has shortcomings in several areas and may therefore be a less useful method than EMG for the detection and prediction of localised muscle fatigue. Other researchers have suggested that the two methods may be used in conjunction to complement each other [151,152].

The ease of application of EMG has led to its application in a various areas in the study of muscle physiology. EMG was detected as early as the 1600s, but its use in the study of muscle physiology has increased since the work of De Luca [35], who greatly influenced the field together with Basmajian. Despite being a popular method in muscle fatigue research, sEMG has some shortcomings. One such shortcoming is related to the signal-to-noise ratio, since the sEMG electrode is susceptible to noise from external factors which may influence the quality of the signal. To overcome this problem, new technology has tried to minimise the noise by classifying signal noise for use in algorithms that will reduce the noise while maximising the sEMG signal. Another important shortcoming of sEMG is, as mentioned above, the placement of the electrode. Electrode placement is important for reliability of the signal, and there has been much discussion on their optimal location. Some studies have suggested certain standards for the placement to ensure repeatability [46] and other studies have suggested the use of different electrodes, including electrode arrays [77].

Most research on sEMG has concentrated on isometric contractions in clinical settings. Studies have analysed the sEMG signal to understand what happens in the signal’s frequency and amplitude when fatigue occurs. Changes in the signal’s frequency and amplitude correlate with the development of fatigue [57,58,67]. The power spectrum density also reveals changes in the sEMG signal, and when fatigue occurs, the frequency content of the signal is compressed proportionally [68].

Recent studies have been conducted on muscle fatigue during dynamic contractions using sEMG for signal acquisition [72]. In dynamic contractions, as well as for isometric contractions, certain changes in the signal’s power spectrum, amplitude and frequency are related to the onset of fatigue [45,73,74]. These findings suggest that sEMG is a reliable method in the study of localised muscle fatigue for both isometric and non-isometric contractions. Although some authors caution against an uncritical use of sEMG as it has several non-obvious limitations [81–83], it has nevertheless been argued that sEMG is the most reliable method for the detection and prediction of localised muscle fatigue [89,90].

When analysing the sEMG signals, feature extraction is an important process which affects the developed application. Traditionally, a range of parameters have been used for the analysis of sEMG signals relating to localised muscle fatigue, such as time domain, frequency domain, and wavelet analysis among others. Recent features have been developed, in particular for the analysis of sEMG signals emanating from fatiguing muscle contractions [77]. Another new trend is to use feature extraction methods to combine features, thus creating composite features, to classify the signal [33,185]. Nevertheless, more work is needed in this area of sEMG research.

Classification is an essential part of the signal analysis of localised muscle fatigue. A range of traditional classification methods are used for sEMG signal analysis but, more interestingly, other classification methods have been used in recent studies on the classification of localised muscle fatigue, such as the fuzzy classifier and GP. Although these are established methods for classification, they have not been used in the area of muscle fatigue research until recently and promising results have been published [7,32,33]. These new methods are important additions in this field, in particular for the development of an autonomous system for detection and prediction of localised muscle fatigue. Nevertheless, more research is needed before the optimal autonomous system can be developed that uses the most appropriate feature extraction and classification model.

## Figures and Tables

**Table 1.** Labelling criteria for the movement aspects.

Movement aspect	Observation
Goniometer entropy	Drop in entropy
Force gauge entropy	Drop in entropy
Goniometer	Vertical belly development of goniometer signal during a trial
Button presses (0–3)(Changed facial/body cues)	Modified Moore-Garg Strain Index

**Table 2.** 10 point scale index.

Scale	Perceived exertion
0	Nothing at all
0.5	Extremely week
1	Very weak
2	Weak
2.5	Moderate
3	”
4	”
5	Strong
6	”
7	Very strong
8	”
9	”
10	Extremely strong

**Table 3.** Adapted Moore-Garg Strain Index, (Bernard, 2001).

Observation	%MVC
Barely noticeable or relaxed effort	5
Noticeable or defined effort	20
Obvious effort; unchanged facial expression	40
Substantial effort; changed facial expression	65
Use of shoulder, trunk or whole body for force application	90

**Table 4.** Modified Moore-Garg Strain index.

Observation	Button presses
Barely noticeable effort	0
Obvious effort (unchanged facial cues)	1
Substantial effort, use of shoulder and changed facial cues	2
Use of shoulder, trunk or whole body for force application	3

**Table 5.** Rule base for signal labelling.

Rules	IF Input 1 (Elbow Angle)	IF Input 2 (Angle Oscillation)	THEN Output
1	Non-Fatigue	Low	Non-Fatigue
2	Non-Fatigue	High	Transition-to-Fatigue
3	Transition-to-Fatigue	Low	Transition-to-Fatigue
4	Transition-to-Fatigue	High	Transition-to-Fatigue
5	Fatigue	Low	Fatigue
6	Fatigue	High	Fatigue
